# The Role of Wearable Devices in Blood Pressure Monitoring and Hypertension Management: A Systematic Review

**DOI:** 10.7759/cureus.75050

**Published:** 2024-12-03

**Authors:** Nikoleta Sinou, Natalia Sinou, Stamatios Koutroulakis, Dimitrios Filippou

**Affiliations:** 1 Biomedical Sciences, National and Kapodistrian University of Athens School of Medicine, Athens, GRC; 2 Medicine, National and Kapodistrian University of Athens School of Medicine, Athens, GRC; 3 Anatomy, National and Kapodistrian University of Athens School of Medicine, Athens, GRC

**Keywords:** applanation tonometry, hypertension, monitoring, plethysmography, wearable devices

## Abstract

Hypertension constitutes a significant risk factor for the development of many coronary artery diseases. In recent years, the advancement of technology and artificial intelligence has led to significant research and breakthroughs in wearable devices that can monitor blood pressure (BP). These devices offer continuous, real-time BP readings, facilitating the early detection and prevention of hypertension. Detailed research was conducted via the PubMed and Scopus databases, using the following keywords: Wearable devices AND Hypertension AND Monitoring. The research was made in the articles from 2017 and subsequently. The aim of the present review is to highlight the benefits of advanced wearable devices, such as smartwatches equipped with BP tracking, inflatable cuffs, finger and wrist monitors, as well as patch and skin-compatible sensors, which provide individuals with the possibility of detecting BP at any time, while also preventing hypertension disease. Finally, wearable devices develop a telehealth connection between patient and physician. However, they ought to be adhered to by particular protocols that attest to their accuracy.

## Introduction and background

Hypertension, often referred to as high blood pressure (BP), is a prevalent modifiable risk factor for coronary artery disease. The elevated tension in arterial walls can cause damage and lead to various cardiovascular issues. Numerous studies have established that the underlying causes of hypertension include vascular resistance, overactivity of the renin-angiotensin-aldosterone system (RAAS), heightened activity of the sympathetic nervous system (SNS), renal dysfunction, compromised endothelial function, and oxidative stress along with inflammation [1]. Additionally, hypertension has both genetic and environmental influences. Stress, depression, and other socioeconomic factors have been recognized as risk factors for hypertension and cardiovascular disease [2].

Recent studies indicate that over 1.28 billion adults worldwide suffer from high BP, particularly in low- and middle-income regions where healthcare is evolving, and in urban settings marked by poor diets, insufficient physical activity, and high stress levels. As previously noted, hypertension is the leading risk factor for cardiovascular diseases [3]. Specifically, elevated BP contributes to heart attacks, atherosclerosis, strokes, heart failure, and kidney impairment. Consequently, effectively managing BP in individuals with hypertension is critically important [4-6].

In recent years, the advancement of technology and artificial intelligence has led to significant research and breakthroughs in wearable devices that can monitor BP. These devices offer continuous, real-time BP readings around the clock, facilitating the early detection and prevention of hypertension. The variety of devices designed for BP monitoring is extensive [1,4]. Research has identified smartwatches equipped with BP tracking, inflatable cuffs, finger and wrist monitors, as well as patch- and skin-compatible sensors [7,8]. Typically, these devices come with enhanced functionalities through smartphones or digital platforms, allowing for data sharing with healthcare providers [9,10]. In particular, we refer to the oscillometric method using devices with a wrist and a finger cuff, in which the point of maximum oscillation during the gradual depressurization of the sphygmomanometer in the arm and the finger cuff, respectively, corresponds to the average intra-arterial pressure. Also, a reference is made to the applanation tonometry method, which is based on the continuous and ongoing monitoring of BP with the use of a device that measures arterial tone, and to the photoplethysmography (PPG) method that is used to identify changes in blood volume in specific body regions during the cardiac cycle. Mobile applications have been designed to identify BP.

This review seeks to explore the spectrum of wearable devices for BP monitoring while emphasizing the significance of their clinical validation. 

## Review

Materials and methods

Search Strategy

The study was conducted during the months of August and September 2024. A thorough investigation was carried out using the published literature found in the PubMed and Scopus databases. The search employed the keywords Wearable devices AND Hypertension AND Monitoring, in both of the two searches. To guarantee precision and completeness, data was collected through a standardized extraction form tailored to these keywords. Information was retrieved using a shared data elicitation form incorporating the specified terms. The research was conducted in accordance with the PRISMA-ScR guidelines (Preferred Reporting Items for Systematic Reviews and Meta-Analyses Extension for Scoping Reviews), which provides a systematic framework for performing scoping reviews (Table 2 of Appendices). All of the authors contributed to the study selection, data extraction, and assessment of the review's quality.

Study Selection

**Figure 1:**
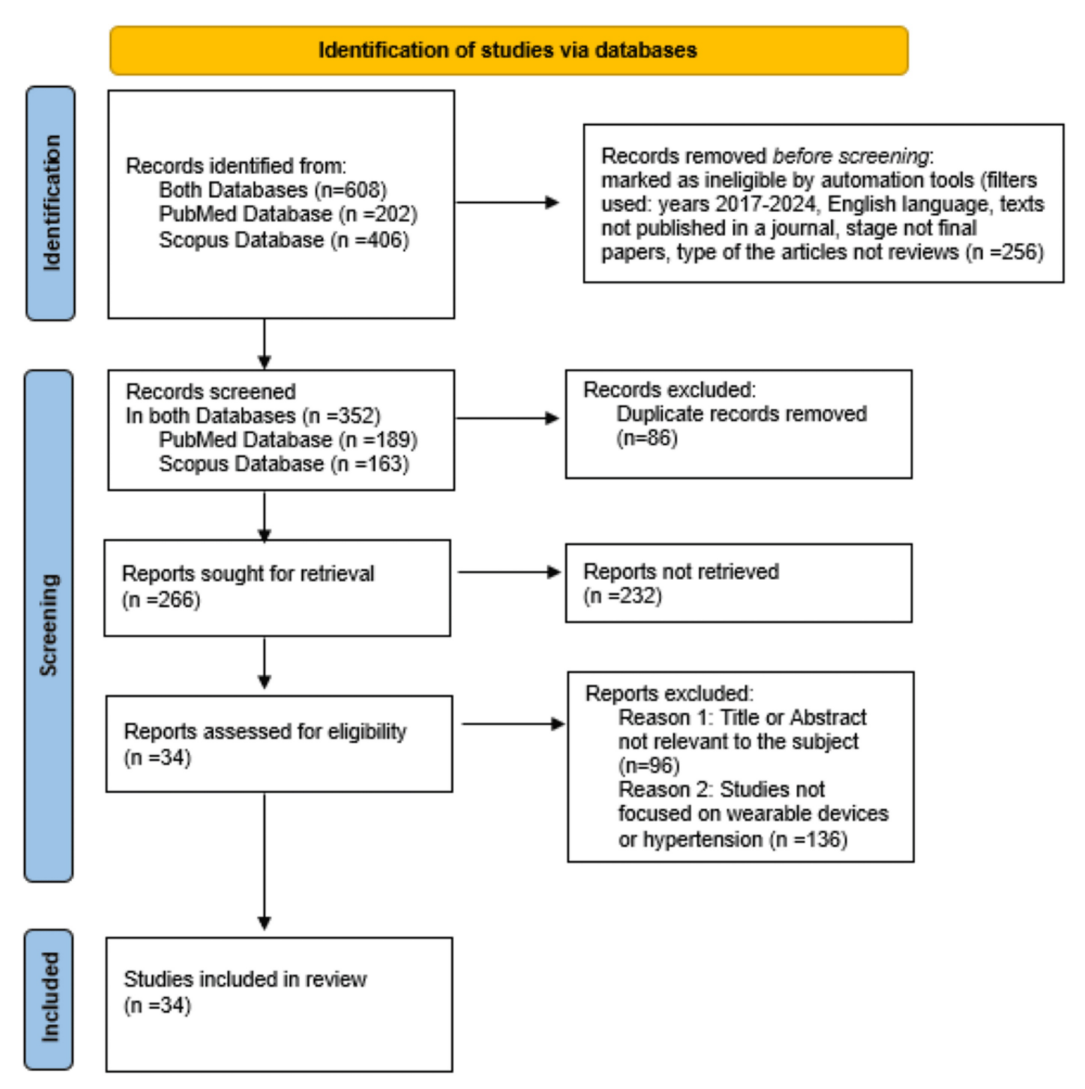
PRISMA diagram PRISMA, Preferred Reporting Items for Systematic Reviews

The studies were carefully selected based on specific criteria that analyzed the development of wearable devices and their role in patients' outcomes. The present systematic review included studies published in journals and final-stage papers. The articles reviewed were of the review type, and the search was conducted on articles published between 2017 and 2024 to ensure they address contemporary scientific issues. According to the exclusion criteria that we used, we did not select articles that were not written in English. Also, studies that did not focus on wearable devices or hypertension were excluded. Thus, the exclusion criteria were: a) text not written in the English language, b) title or abstract not relevant to the subject, c) study not focused on wearable devices or hypertension, and d) Abstract not available in PubMed or Scopus database. The inclusion criteria were: a) studies published in a journal, b) final-stage papers, c) research papers and only selected systematic reviews and meta-analyses, possessing particular interest and referring mainly to specific guidelines, and d) articles from 2017 until now. In accordance with the PRISMA guidelines, a total of 202 records were initially identified through a search on PubMed, while 406 records were retrieved from the Scopus database, resulting in a total of 608 records. A total of 256 records were marked as ineligible by automation tools and removed prior to screening. The filters applied included publication years (2017-2024), English language, exclusion of texts not published in journals, exclusion of papers not in their final stage, and exclusion of articles that were not reviews. Thus, in total, 352 records were screened (189 in PubMed and 163 in Scopus database). After a thorough screening, the duplicates between the records selected from PubMed and Scopus databases were 86. After removing the duplicates, the final number of records sought for retrieval in both databases was 266. Finally, 34 reports were assessed for eligibility, resulting in the exclusion of 232 articles. The exclusions were due to two main reasons: 96 articles had titles or abstracts not relevant to the subject, and 136 studies did not focus on wearable devices or hypertension, rendering them unrelated to the research. Thus, this specific article is founded on data sourced from 34 credible references (Figure 1).

Literature Screening

A thorough technique was used to evaluate the data according to the classification of the data mentioned above. While developing the themes, we took into consideration the usage of wearable technology and its effect on patient outcomes, as they are presented in all of the articles we selected as citations. In Table 1, we summarize the characteristics of the included articles. The literature screening of the articles was conducted by two authors independently.

**Table 1:** Characteristics of the included articles PPG, photoplethysmography

Study	Wrist cuff method	Finger cuff method	Applanation tonometry method	PPG method	Applications in smartphones with PPG integrated
Schutte AE, et al., 2022 [1]	Yes	Yes	Not mentioned	Not mentioned	Not mentioned
Zhang Y, et al., 2020 [2]	Yes	Yes	Not mentioned	Yes	Yes
Pan HY, et al., 2024 [3]	Yes	Yes	Yes	Yes	Yes
Kario K, 2023 [4]	Yes	Yes	Not mentioned	Yes	Yes
Han M, et al., 2023 [5]	Yes	Yes	Not mentioned	Not mentioned	Yes
Gauer RL, et al., 2024 [6]	Yes	Yes	Not mentioned	Yes	Not mentioned
Pandit JA, et al., 2020 [7]	Yes	Yes	Yes	Yes	Yes
Kario K, 2017 [8]	Yes	Yes	Not mentioned	Not mentioned	Not mentioned
Marvasti TB, et al., 2024 [9]	Yes	Yes	Not mentioned	Yes	Yes
Stergiou GS, et al., 2023 [10]	Yes	Yes	Not mentioned	Yes	Not mentioned
Zheng Y, et al., 2019 [11]	Yes	Not mentioned	Not mentioned	Yes	Not mentioned
Yuting Z, et al., 2023 [12]	Yes	Yes	Not mentioned	Not mentioned	Yes
Stergiou GS, et al., 2022 [13]	Yes	Yes	Not mentioned	Not mentioned	Yes
Scalise F, et al., 2020 [14]	Not mentioned	Not mentioned	Not mentioned	Yes	Not mentioned
Lee Y, et al., 2018 [15]	Not mentioned	Not mentioned	Not mentioned	Not mentioned	Yes
Gnanenthiran SR, et al., 2023 [16]	Yes	Yes	Not mentioned	Not mentioned	Not mentioned
Rosa D, et al., 2024 [17]	Yes	Not mentioned	Not mentioned	Not mentioned	Yes
Moon JH, et al., 2020 [18]	Yes	yes	Not mentioned	Yes	Yes
Islam SMS, et al., 2022 [19]	Yes	Yes	Not mentioned	Yes	Yes
Kario K, 2020 [20]	Yes	Yes	Yes	Yes	Yes
Konstantinidis D, et al., 2022 [21]	Yes	Yes	Yes	Yes	Yes
Elgendi M, et al., 2019 [22]	Yes	Yes	Yes	Yes	Yes
Franklin D, et al., 2023 [23]	Not mentioned	Not mentioned	Not mentioned	Yes	Not mentioned
Zhang Q, et al., 2017 [24]	Yes	Yes	Not mentioned	Yes	Yes
Ganti VG, et al., 2021 [25]	Not mentioned	Not mentioned	Not mentioned	Yes	Yes
Park SH, et al., 2019 [26]	Yes	Yes	Not mentioned	Yes	Not mentioned
Allinson F, et al., 2024 [27]	Yes	Not mentioned	Not mentioned	Yes	Yes
Lobo EH, et al., 2023 [28]	Yes	Yes	Not mentioned	Not mentioned	Yes
Kario K, et al., 2020 [29]	Yes	Yes	Not mentioned	Not mentioned	Not mentioned
Welykholowa K, et al., 2020 [30]	Yes	Yes	Yes	Yes	Not mentioned
Georgi N, et al., 2019 [31]	Yes	Yes	Not mentioned	Not mentioned	Yes
Chandrasekaran R, et al., 2023 [32]	Yes	Yes	Not mentioned	Not mentioned	Not mentioned
Mukkamala R, et al., 2021 [33]	Yes	Not mentioned	Not mentioned	Not mentioned	Not mentioned
Spatz ES, et al., 2024 [34]	Yes	Yes	Not mentioned	Yes	Yes

Results

We conducted a thorough search of 34 articles published between 2017 and 2024 using the PubMed and Scopus databases. Following a comprehensive review of all the referenced articles, we found that traditional BP measurement methods are increasingly being supplanted by sophisticated wearable monitoring devices. Notably, all of the articles indicated that conventional techniques, such as the auscultatory and oscillometric methods, fall short of providing continuous 24-hour measurements and rely on specific pressure readings. The literature consistently emphasizes that these advanced wearable devices have taken over from traditional methods, allowing for the monitoring of patients' BP during daily activities, sleep, and stressful situations, and in some cases, notifying patients of abnormal readings in relation to their cardiovascular conditions. Specifically, 38 of the studies point to the oscillometric technique utilizing wrist and finger cuff devices as an alternative to traditional methods. 25 sources advocate for the use of PPG, a non-invasive optical technique that detects changes in blood volume in specific body areas throughout the cardiac cycle. Applanation tonometry is recommended by seven articles as a non-invasive approach to measure the force required to flatten a superficial artery. Finally, 24 sources emphasize that novel data analysis techniques have unveiled new technologies and telecommunication systems, which could enhance algorithms in wearable devices, offering more tailored monitoring and interventions for cardiovascular health. According to a thorough study of all of our references, the method of using a wrist and an arm cuff for BP measuring was first introduced in 2002. Since 2010, many studies have been conducted that highlight the BP self-measurement, with different methods.

Discussion

Traditional Methods of Measuring Blood Pressure

A standard BP monitor utilizes auscultatory and oscillometric techniques, both involving a cuff. The cuff is wrapped around the patient's arm and inflated until the external pressure exceeds the systolic pressure of the artery, thereby halting blood flow beneath it [1]. The stethoscope is placed on the brachial artery. BP is measured when the physician listens to Korotkoff sounds using a stethoscope (Auscultatory method). In the oscillometric approach, arterial pressure is indicated by the peak oscillations, and an algorithm is used to determine systolic and diastolic pressures. Despite being the most widely used method for measuring BP, it has several limitations. Cuff bladder monitoring devices are unable to provide continuous, 24-hour pressure readings since the measurements must be taken manually. Additionally, a one- to two-minute interval is essential for the patient’s hemodynamics to stabilize after being under pressure. Furthermore, the accuracy of these measurements is significantly influenced by the cuff inflation and the effort exerted by the healthcare professional [11,12]. An alternative method for measuring BP is arterial catheterization, but this approach is invasive and intended solely for use in a hospital setting [11,13]. 

Advanced Wearable Monitoring Devices

Advanced wearable devices that monitor BP have been replacing traditional methods, offering patients continuous, accurate, and user-friendly BP tracking as a result of the advancement of technology and the numerous clinical trials that have been conducted [7,14]. 

In particular, these devices provide around-the-clock monitoring, capturing BP during daily activities, periods of stress, rest, and sleep, enabling the identification of any concealed spikes. This innovative approach is designed, additionally, to collect and store all the measured data, leading to a more comprehensive overview of a patient's readings by the doctors [3,8]. Furthermore, some of these advanced wearable devices can prevent patients from developing dangerously high BP by identifying specific alerting readings, based on the patient's medical history, that may elevate cardiovascular risk. Even more sophisticated models enable proactive interventions in response to this risk [15-17].

Oscillometric Method Using Devices With Wrist Cuff

As mentioned previously, the fundamental idea behind the oscillometric method is that the point of maximum oscillation during the gradual depressurization of the sphygmomanometer in the cuff corresponds to the average intra-arterial pressure [6]. The primary drawback of this method is its inability to accurately measure BP in relation to physical activity. The sphygmomanometer's readings are disrupted by noise caused by the patient‘s movement, especially when the patient is exercising [2]. More sophisticated devices employing the same technique are designed to compress the muscles less. According to some studies, an innovative clock-sized BP measuring device uses an inflatable belt and is based on the oscillometric method. Specifically, this device comes in two sizes, is used on a regular basis, and measures the patient's body during exercise, stress, and even sleep. This device is comfortable to use and has been approved to take a patient's BP accurately when the wrist is at the level of the heart. It is expected that this device will assess measurements regardless of wrist posture during ongoing clinical testing [16-18].

Oscillometric Method Using Devices With Finger Cuff

The oscillometric technique is widely used in wrist cuff devices for measuring BP. The finger cuff is specifically fitted around the patient's finger and inflated to a level that stops blood flow [2]. A pressure sensor built into the cuff measures the pressure within the cuff, as the cuff gradually deflates. The oscillations correlate to the diastolic and systolic BP; the diastolic oscillations can be identified by the point at which they begin to fall as the cuff continues to deflate, and the systolic oscillations may be identified by the point at which they begin to climb [2,17,19].

Devices for finger cuffs offer benefits regarding portability and size. Specifically, they are smaller than traditional arm cuffs and portable, which makes them more practical for individual use [7]. However, their measurements are influenced by both the position of the arm cuff and the finger, making them less precise than arm cuffs. Additionally, the temperature, stress, or movement of the finger can interfere with monitoring [7]. 

Applanation Tonometry Method

This approach, initially used in 1963, is based on the continuous and ongoing monitoring of BP with the use of a device that measures arterial tone. The force needed to flatten a superficial artery can be measured noninvasively using arterial tonometry [19]. More precisely, the pressure required to accomplish this flattery equals this of the BP. Because of its accessibility, great diameter, shallow depth beneath the skin, and attachment to the radial bone, the artery at the wrist is the most frequently used. The sensor must remain stationary and squeezed on the artery throughout the measurements [19]. The device's probe flattens a section of the artery wall by applying gentle yet constant pressure to the skin. Tension transducers that come into touch with the artery wall measure the applied pressure, and the measurement represents accurately the arterial pressure [20]. The transducer measures the endo-aortic pressure while the arterial wall remains unaltered. The oscillometric approach though measures the diastolic and systolic pressures while the artery gradually decompresses [19,20].

Two benefits of arterial tonometry are the ability to directly measure arterial pressure and the possibility of ongoing monitoring with real-time data. However, this approach does have certain drawbacks [1,21]. The sensor's position determines the variation in the measurement site but should remain constant throughout the measurements. The measurements are more accurate when the patient is seated and not standing [28]. Additionally, the method‘s accuracy is also being examined since it relies on the location of the artery and the force needed to compress it. Another drawback is that the measurements are related to the position between the heart and the monitor as it affects the levels of hydrostatic pressure [21].

A strong interest has been shown by patients with sleep apnea syndrome in devices that employ the applanation tonometry method [1,22]. This technique has specifically been applied to the detection of nocturnal BP and BP surges. Through numerous clinical studies, an algorithm for identifying BP peaks has been developed; the algorithm is based on the BP readings taken throughout the night [22].

Photoplethysmography Method

PPG, a non-invasive optical technique, first investigated in 1930, is used to identify changes in blood volume in specific body regions during the cardiac cycle [23]. Specifically, it is a technique that gauges how much light is reflected or absorbed by blood vessels. A light source, either green or infrared, is positioned on the edge, usually on the finger. The amount of light reflected back by the skin is measured by a photoprotector, which then captures the reflected light and transforms it into an electrical signal [1,6]. Since the amount of light absorbed or reflected is proportional to the volume of blood in the vessel, PPG considers variations in blood volume rather than BP [20]. This technique creates the PPG signal by detecting and recording changes in blood volume to the sensor. The sensor also takes into account capillaries, veins, and arteries. A waveform that represents the cardiac cycle is produced by the reflected signals that the device picks up from the light that is continuously emitted onto it [9,10,21].

The time it takes for a pulse wave produced by the heartbeat to move between two arterial sites is known as the pulse transit time (PTT) [20,22,23]. More precisely, during the ventricular explosion, a pulse pressure wave is created, as the tension in the arterial wall is bigger than the velocity of the blood. PTT calculates how long it takes for a pulse wave produced by a heartbeat to reach a peripheral artery [22,24]. The ECG and PPG are the two common techniques used to measure PTT. Specifically, PPG measures the variations in blood volume in numerous tissues throughout the cardiac cycle, and ECG serves as a time provider. In other words, the PPG measures the duration of a pulse wave between the heart and the finger (peripheral site), whereas the ECG detects the R-wave or the heartbeat's peak. By measuring the interval between the R-wave and the pulse wave's arrival at the peripheral site, PTT allows us to determine BP levels [20,22,25,26].

As a result of numerous clinical studies and experiments, devices with PPG sensors that are integrated into wearable sensors, like wrist-sized devices (smartwatches), have been created by several companies. There are many clinical benefits; for example, these devices can currently measure physiological parameters such as body temperature, heart rate, BP, and physical activity [21]. However, there are still certain limitations in these devices regarding their sensitivity to body temperature, vascular health, and movement. Furthermore, the device should be calibrated continuously due to people’s variations in heart rates [21,26].

Applications of Smartphones With Integrated Photoplethysmography

An innovative method that combines the ability to measure BP non-invasively and continuously with the use of smartphones is the integration of PPG into smartphone applications for hypertension monitoring [20,27]. The effectiveness of using PPG signals to monitor BP has been demonstrated in numerous clinical studies. The PPG sensors, which are required for the measurements, are found in smartphones' cameras, flashlights, and external sensors that are connected to the device via Bluetooth or USB [21,22,24]. Additionally, an algorithm must be developed to gather PPG signals, extract relevant cardiovascular metrics such as heart rate and PTT, and measure BP. The algorithm specifically recognizes, evaluates, and transforms PPG waveforms into cardiovascular metrics. Each measure can be collected and saved for long-term tracking by the mobile application that is installed on people's smartphones. When monitoring pathological BP readings that exceed physiological recommendations, the software can be programmed to notify users [28-30]. There are numerous benefits that this application offers people. Specifically, without the need for specialized medical equipment, they are able to take BP readings at any time and from any location. Because the measurements can be recorded, people have the opportunity to gather data continuously and share it with physicians [29]. Nevertheless, there are still certain restrictions on the application's use in terms of its effectiveness because temperature, movement, camera angle, and environmental factors can all affect measurements [33].

Potentials for the Future

Doctors can now monitor their patients' BP thanks to new technologies and telecommunication systems that have been made available by new data analysis techniques [31]. Specifically, medical professionals can know a patient's measurements when they are not in the office and treat them accordingly without the patient having to see them. In addition to telehealth integration, wearable technology can give users access to their individual profiles, data, and measurements, allowing them to continuously and sequentially review their measurements and track any cardiovascular conditions [13,19]. There is still a necessity to develop artificial intelligence that provides summary reports of this enormous data collection and notifies doctors or specialized nurses of warnings such as hypertensive urgencies [17,27].

There are certain limitations, though, even if these devices offer a more fruitful patient-provider relationship. First and foremost, they ought to be adhered to by particular protocols that attest to their accuracy. With their unreliable measures, many of the developed applications can easily mislead people and are not certified by medical protocols [21,31,32]. Furthermore, several factors, including arterial BP, heart rate, oxygen saturation, stroke volume, LDL, and glucose levels, among others, affect a patient's cardiovascular health [22]. Additionally, the patient's fear or anxiety about receiving high or even low BP measurements may affect their overall well-being. Only BP can be measured by the aforementioned wearable technology [13,22,33,34].

Therefore, due to the potential these technologies offer, integrating telehealth and creating a unique profile for each individual may serve as supplementary strategies to standard medical procedures. However, these devices cannot take the place of routine medical visits, and the relationship between patient and doctor is irreplaceable because only a doctor can be able to combine, overview, and analyze a patient’s history and advise them suitably [13,22].

## Conclusions

Advancements in technology and artificial intelligence have led to the development of innovative wearable devices that can detect and monitor BP. These devices provide patients with continuous, user-friendly, and accurate blood pressure tracking, record measurements, and create a personalized profile that can be shared with physicians anytime, even outside of the office. Moreover, they can alert patients when a pathological measure is detected. However, wearable devices must adhere to specific protocols that ensure their accuracy and safety. Finally, further advancements in the technology behind wearable devices are needed to provide a more personalized and comprehensive range of medical parameters for clinical use.

## Appendices

**Table 2:** PRISMA 2020 checklist PRISMA, Preferred Reporting Items for Systematic Reviews

Section and Topic	Item #	Checklist item	Location where item is reported
TITLE			
Title	1	Identify the report as a systematic review.	1^st^ page
ABSTRACT			
Abstract	2	See the PRISMA 2020 for Abstracts checklist.	1^st^ paragraph
INTRODUCTION			
Rationale	3	Describe the rationale for the review in the context of existing knowledge.	1^st^-3^rd^ paragraph of introduction
Objectives	4	Provide an explicit statement of the objective(s) or question(s) the review addresses.	4^th^ paragraph of introduction
METHODS			
Eligibility criteria	5	Specify the inclusion and exclusion criteria for the review and how studies were grouped for the syntheses.	2^nd^ paragraph of materials and methods (study selection)
Information sources	6	Specify all databases, registers, websites, organizations, reference lists, and other sources searched or consulted to identify studies. Specify the date when each source was last searched or consulted.	1^st^ paragraph of materials and methods (search strategy)
Search strategy	7	Present the full search strategies for all databases, registers, and websites, including any filters and limits used.	1^st^-2^nd^ paragraph of materials and methods (search strategy-study selection)
Selection process	8	Specify the methods used to decide whether a study met the inclusion criteria of the review, including how many reviewers screened each record and each report retrieved, whether they worked independently, and if applicable, details of automation tools used in the process.	2^nd^-3^rd^ paragraph of materials and methods (study selection-literature screening)
Data collection process	9	Specify the methods used to collect data from reports, including how many reviewers collected data from each report, whether they worked independently, any processes for obtaining or confirming data from study investigators, and if applicable, details of automation tools used in the process.	1^st^ paragraph of materials and methods (search strategy)
Data items	10a	List and define all outcomes for which data were sought. Specify whether all results that were compatible with each outcome domain in each study were sought (e.g., for all measures, time points, analyses), and if not, the methods used to decide which results to collect.	2^nd^ paragraph of materials and methods (study selection)
	10b	List and define all other variables for which data were sought (e.g., participant and intervention characteristics, funding sources). Describe any assumptions made about any missing or unclear information.	2^nd^ paragraph of materials and methods (study selection)
Study risk of bias assessment	11	Specify the methods used to assess risk of bias in the included studies, including details of the tool(s) used, how many reviewers assessed each study, and whether they worked independently, and if applicable, details of automation tools used in the process.	2^nd^ paragraph of materials and methods (study selection)
Effect measures	12	Specify for each outcome the effect measure(s) (e.g., risk ratio and mean difference) used in the synthesis or presentation of results.	2^nd^ paragraph of materials and methods (study selection)
Synthesis methods	13a	Describe the processes used to decide which studies were eligible for each synthesis (e.g., tabulating the study intervention characteristics and comparing against the planned groups for each synthesis (item #5)).	2^nd^ paragraph of materials and methods (study selection)
	13b	Describe any methods required to prepare the data for presentation or synthesis, such as handling of missing summary statistics or data conversions.	2^nd^ paragraph of materials and methods (study selection)
	13c	Describe any methods used to tabulate or visually display results of individual studies and syntheses.	3^rd^ paragraph of materials and methods (literature screening)
	13d	Describe any methods used to synthesize results and provide a rationale for the choice(s). If meta-analysis was performed, describe the model(s), method(s) to identify the presence, and extent of statistical heterogeneity, and software package(s) used.	3^rd^ paragraph of materials and methods (literature screening)
	13e	Describe any methods used to explore possible causes of heterogeneity among study results (e.g., subgroup analysis and meta-regression).	1^st^-2^nd^ paragraph of materials and methods (search strategy-study selection)
	13f	Describe any sensitivity analyses conducted to assess robustness of the synthesized results.	None
Reporting bias assessment	14	Describe any methods used to assess risk of bias due to missing results in a synthesis (arising from reporting biases).	2^nd^ paragraph of materials and methods (study selection)
Certainty assessment	15	Describe any methods used to assess certainty (or confidence) in the body of evidence for an outcome.	3^rd^ paragraph of materials and methods (literature screening)
RESULTS			
Study selection	16a	Describe the results of the search and selection process, from the number of records identified in the search to the number of studies included in the review, ideally using a flow diagram.	1^st^ paragraph of results
	16b	Cite studies that might appear to meet the inclusion criteria, but which were excluded, and explain why they were excluded.	1^st^ paragraph of results
Study characteristics	17	Cite each included study and present its characteristics.	1^st^ paragraph of results
Risk of bias in studies	18	Present assessments of risk of bias for each included study.	1^st^ paragraph of results
Results of individual studies	19	For all outcomes, present, for each study: (a) summary statistics for each group (where appropriate) and (b) an effect estimate and its precision (e.g., confidence/credible interval), ideally using structured tables or plots.	1^st^ paragraph of results
Results of syntheses	20a	For each synthesis, briefly summarize the characteristics and risk of bias among contributing studies.	1^st^ paragraph of results
	20b	Present results of all statistical syntheses conducted. If meta-analysis was done, present for each the summary estimate and its precision (e.g., confidence/credible interval) and measures of statistical heterogeneity. If comparing groups, describe the direction of the effect.	1^st^ paragraph of results
	20c	Present results of all investigations of possible causes of heterogeneity among study results.	1^st^ paragraph of results
	20d	Present results of all sensitivity analyses conducted to assess the robustness of the synthesized results.	1^st^ paragraph of results
Reporting biases	21	Present assessments of risk of bias due to missing results (arising from reporting biases) for each synthesis assessed.	1^st^ paragraph of results
Certainty of evidence	22	Present assessments of certainty (or confidence) in the body of evidence for each outcome assessed.	1^st^ paragraph of results
DISCUSSION			
Discussion	23a	Provide a general interpretation of the results in the context of other evidence.	1-13^th^ paragraph of discussion
	23b	Discuss any limitations of the evidence included in the review.	14^th^ paragraph of discussion (Potentials for the future)
	23c	Discuss any limitations of the review processes used.	No limitations of the review
	23d	Discuss implications of the results for practice, policy, and future research.	13^th^ paragraph of discussion
OTHER INFORMATION			
Registration and protocol	24a	Provide registration information for the review, including register name and registration number, or state that the review was not registered.	1^st^ page (correspondence address)
	24b	Indicate where the review protocol can be accessed, or state that a protocol was not prepared.	No protocol prepared
	24c	Describe and explain any amendments to information provided at registration or in the protocol.	No amendments
Support	25	Describe sources of financial or non-financial support for the review and the role of the funders or sponsors in the review.	No sources of financial
Competing interests	26	Declare any competing interests of review authors.	No competing interests
Availability of data, code, and other materials	27	Report which of the following are publicly available and where they can be found: template data collection forms; data extracted from included studies; data used for all analyses; analytic code; any other materials used in the review.	None

## References

[1] Schutte AE, Kollias A, Stergiou GS (2022). Blood pressure and its variability: classic and novel measurement techniques. Nat Rev Cardiol.

[2] Zhang Y, Fang Y, Xu Y (2020). Adherence with blood pressure monitoring wearable device among the elderly with hypertension: the case of rural China. Brain Behav.

[3] Pan HY, Lee CK, Liu TY, Lee GW, Chen CW, Wang TD (2024). The role of wearable home blood pressure monitoring in detecting out-of-office control status. Hypertens Res.

[4] Kario K (2023). Digital hypertension towards to the anticipation medicine. Hypertens Res.

[5] Han M, Lee YR, Park T (2023). Feasibility and measurement stability of smartwatch-based cuffless blood pressure monitoring: A real-world prospective observational study. Hypertens Res.

[6] Gauer RL, Thomas MF, McNutt RA (2024). Palpitations: evaluation, management, and wearable smart devices. Am Fam Physician.

[7] Pandit JA, Lores E, Batlle D (2020). Cuffless blood pressure monitoring: promises and challenges. Clin J Am Soc Nephrol.

[8] Kario K (2017). Perfect 24-h management of hypertension: clinical relevance and perspectives. J Hum Hypertens.

[9] Marvasti TB, Gao Y, Murray KR, Hershman S, McIntosh C, Moayedi Y (2024). Unlocking tomorrow’s health care: expanding the clinical scope of wearables by applying artificial intelligence. Can J Cardiol.

[10] Stergiou GS, Avolio AP, Palatini P (2023). European Society of Hypertension recommendations for the validation of cuffless blood pressure measuring devices: European Society of Hypertension working group on blood pressure monitoring and cardiovascular variability. J Hypertens.

[11] Liang Y, Abbott D, Howard N, Lim K, Ward R, Elgendi M (2019). How effective is pulse arrival time for evaluating blood pressure? Challenges and recommendations from a study using the mimic database. J Clin Med.

[12] Yuting Z, Xiaodong T, Qun W (2023). Effectiveness of a mHealth intervention on hypertension control in a low-resource rural setting: a randomized clinical trial. Front Public Health.

[13] Stergiou GS, Mukkamala R, Avolio A (2022). Cuffless blood pressure measuring devices: review and statement by the European Society of Hypertension working group on blood pressure monitoring and cardiovascular variability. J Hypertens.

[14] Scalise F, Margonato D, Sole A, Sorropago A, Sorropago G, Mancia G (2020). Ambulatory blood pressure monitoring by a novel cuffless device: a pilot study. Blood Press.

[15] Lee Y, Howe C, Mishra S (2018). Wireless, intraoral hybrid electronics for real-time quantification of sodium intake toward hypertension management. Proc Natl Acad Sci.

[16] Gnanenthiran SR, Tan I, Atkins ER (2023). Transforming blood pressure control in primary care through a novel remote decision support strategy based on wearable blood pressure monitoring: the NEXTGEN-BP randomized trial protocol. Am Heart J.

[17] Rosa D, Peverelli M, Poliani A, Villa G, Manara DF (2024). Exploring hypertension patient engagement using mHealth. A scoping review. High Blood Press Cardiovasc Prev.

[18] Moon JH, Kang MK, Choi CE, Min J, Lee HY, Lim S (2020). Validation of a wearable cuff-less wristwatch-type blood pressure monitoring device. Sci Rep.

[19] Islam SM, Chow CK, Daryabeygikhotbehsara R (2022). Wearable cuffless blood pressure monitoring devices: a systematic review and meta-analysis. Eur Heart J Digit Health.

[20] Kario K (2020). Management of hypertension in the digital era: small wearable monitoring devices for remote blood pressure monitoring. Hypertension.

[21] Konstantinidis D, Iliakis P, Tatakis F, Thomopoulos K, Dimitriadis K, Tousoulis D, Tsioufis K (2022). Wearable blood pressure measurement devices and new approaches in hypertension management: the digital era. J Hum Hypertens.

[22] Elgendi M, Fletcher R, Liang Y (2019). The use of photoplethysmography for assessing hypertension. NPJ Digit Med.

[23] Franklin D, Tzavelis A, Lee JY (2023). Synchronized wearables for the detection of haemodynamic states via electrocardiography and multispectral photoplethysmography. Nat Biomed Eng.

[24] Zhang Q, Zhou D, Zeng X (2017). Highly wearable cuff-less blood pressure and heart rate monitoring with single-arm electrocardiogram and photoplethysmogram signals. Biomed Eng Online.

[25] Ganti VG, Carek AM, Nevius BN, Heller JA, Etemadi M, Inan OT (2021). Wearable cuff-less blood pressure estimation at home via pulse transit time. IEEE J Biomed Health Inform.

[26] Park SH, Zhang Y, Rogers JA, Gallon L (2019). Recent advances of biosensors for hypertension and nephrology. Curr Opin Nephrol Hypertens.

[27] Allinson F, Mejia N, Ariniello L, Quer G, Muse ED (2024). A pilot study exploring novel contexts for out-of-office blood pressure measurement. Front Cardiovasc Med.

[28] Lobo EH, Karmakar C, Abdelrazek M (2023). Design and development of a smartphone app for hypertension management: an intervention mapping approach. Front Public Health.

[29] Kario K, Shimbo D, Tomitani N, Kanegae H, Schwartz JE, Williams B (2020). The first study comparing a wearable watch-type blood pressure monitor with a conventional ambulatory blood pressure monitor on in-office and out-of-office settings. J Clin Hypertens (Greenwich).

[30] Welykholowa K, Hosanee M, Chan G (2020). Multimodal photoplethysmography-based approaches for improved detection of hypertension. J Clin Med.

[31] Georgi N, Corvol A, Le Bouquin Jeannès R (2019). For a more reliable measure of wrist blood pressure using smartwatch. Telemed J E Health.

[32] Chandrasekaran R, Sharma P, Moustakas E (2023). Exploring disparities in healthcare wearable use among cardiovascular patients: findings from a national survey. Rev Cardiovasc Med.

[33] Mukkamala R, Yavarimanesh M, Natarajan K, Hahn JO, Kyriakoulis KG, Avolio AP, Stergiou GS (2021). Evaluation of the accuracy of cuffless blood pressure measurement devices: challenges and proposals. Hypertension.

[34] Spatz ES, Ginsburg GS, Rumsfeld JS, Turakhia MP (2024). Wearable digital health technologies for monitoring in cardiovascular medicine. N Engl J Med.

